# Growth, Water Use, and Nitrate-^15^N Uptake of Greenhouse Tomato as Influenced by Different Irrigation Patterns, ^15^N Labeled Depths, and Transplant Times

**DOI:** 10.3389/fpls.2017.00666

**Published:** 2017-05-02

**Authors:** Maomao Hou, Qiu Jin, Xinyu Lu, Jiyu Li, Huizhen Zhong, Yue Gao

**Affiliations:** ^1^College of Horticulture, Fujian Agriculture and Forestry UniversityFuzhou, China; ^2^Institute of Water Conservancy Science of Jiangsu ProvinceNanjing, China; ^3^Development and Reform Commission of SuihuaSuihua, China

**Keywords:** *Solanum lycopersicum L*, partial root-zone irrigation, water use efficiency, N recovery, N loss, quality, yield

## Abstract

Increasing water use efficiency and reducing nitrogen pollutant discharge are important tasks for modern agriculture. To evaluate the effect of alternate partial root-zone irrigation (APRI) on tomato plant growth, water use efficiency and nitrate-^15^N uptake, an experiment was conducted from June to December in 2014 under greenhouse condition in northern China. The experiment contained two irrigation patterns [APRI and conventional irrigation (CI)], two ^15^N labeled depths in soil (10 and 50 cm) and two transplant time (early and late summer). Results showed that, compared to CI, APRI did not significantly (*p* > 0.05) impact the growth and biomass accumulation in aboveground part of tomato, while it enhanced the root, reflecting by greater length density, and more dry mass. APRI produced marginally lower yields, but saved 34.9% of irrigation water, and gave a 37.6–49.9% higher water use efficiency relative to CI. In addition, APRI improved fruit quality, mainly through increasing the contents of soluble solid (by 12.8–21.6%), and vitamin C (2.8–12.7%), and the sugar/acid ratio (3.5–8.5%). The ^15^N utilization efficiency (^15^NUE) in APRI was higher than that in CI, which was more evident when ^15^N was labeled at 50 cm depth. Significant (*p* < 0.05) ^15^N recovery increase of 10.2–13.2% and ^15^N loss decrease of 35.4–54.6% were found for APRI compared to CI. The overall results suggest that APRI under greenhouse could benefit the nitrate-N recovery and increase the water use efficiency in tomato.

## Introduction

Greenhouse agriculture achieves great success in many countries like Netherlands (Korthals Altes and van Rij, 2013), Israel (Teitel and Zhao, 1992; Elad et al., 2014), Japan (Kinoshita et al., 2016), and the United States (Burnett et al., 2016). China has the world's largest area of greenhouse agriculture, however, more than 90% of the greenhouses use primitive facilities, and soil culture is still the main method for crop production (Du, 2007). In a long time, the purpose of China's vegetable production is to acquire high yield, the energy, water and fertilizer resources are seriously overused, leading to a serious waste of agricultural inputs. A survey has shown that in China, average inorganic N input for one season vegetable under greenhouse is 569–2,000 kg/ha, which is several times or even dozen times over that applied to field crop, quantities of the applied fertilizer nitrogen are residual in the soil (Dorais et al., 2005).

The residual inorganic N in dryland soil is mainly existed in nitrate nitrogen (${\text{NO}}_{3}^{-}$-N) form, which is difficult to be absorbed by soil particles (Wang X. et al., 2015), and is easy to migrate downward along with the irrigation water (Kanthle et al., 2016). Due to the weak denitrification effect, ${\text{NO}}_{3}^{-}$-N in the deep soil layer is hard to transform to other N forms, it will move to the deeper soil layer and pose a threat to the underground water environment (Stefanelli et al., 2010). ${\text{NO}}_{3}^{-}$-N leaching is influenced by various factors, the vertical movement of soil water is one of the most important factors that affecting the distribution of ${\text{NO}}_{3}^{-}$-N in profile soil (Wallis et al., 2011). Some studies have employed innovative irrigation methods to change the ${\text{NO}}_{3}^{-}$-N location and the crop ${\text{NO}}_{3}^{-}$-N uptake (Sepaskhah and Tafteh, 2012; Liu et al., 2015; Wang X. et al., 2015).

In recent years, alternate partial root-zone irrigation (APRI) has been shown to be an effective irrigation technique in many regions of the world (Wei et al., 2016). APRI irrigates only part of the root zone leaving the other part to dry to a predetermined level before the next irrigation, it is a further development of deficit irrigation (Wang et al., 2012). APRI is found to improve water productivity of crop production without much yield reduction (Dodd, 2009; Du et al., 2015) including in tomato (Sarker et al., 2016; Wei et al., 2016). The APRI-induced plant responses include reduced leaf initiation and expansion rate and decreased inefficient transpiration but without significant reduction in photosynthesis, thus increasing the intrinsic water use efficiency (WUE) (Wei et al., 2016). Sezen et al. (2011) conducted a 2-year experiment in the eastern Mediterranean region of Turkey and revealed that partial root-zone irrigation is acceptable for sunflower production under a water scarcity situation, which received about 36% less irrigation water, reduced the yield by 15%, but resulted in a dramatically high WUE of 1.0 kg m^−3^. Topak et al. (2016) study in semi-arid area demonstrated that, APRI with 50% full irrigation water increased the root WUE of sugar beet by 19.8% compared to full irrigation, and by 8.5% compared to conventional deficit irrigation with a same amount of applied water. Moreover, the root dry biomass is found higher in APRI plants than that in conventional deficit irrigation and full irrigation plants (Wang et al., 2012). One reason is that plants may effectively forage for water in APRI treatment by proliferating their roots into the wetted root-zones (Du et al., 2008), and the other reason is that alternating the wet and dry parts of the root-zone stimulates growth of the previously dry root system (Mingo et al., 2004). In addition, Sun (Sun et al., 2013) reported that APRI significantly increases plant N content in relation to the conventional deficit irrigation practice. However, when using 50% of the full irrigation amount, APRI showed significant yield decrease (by 52%) in processing tomato, according to Casa and Rouphael's research (Casa and Rouphael, 2014). Although, many positive or negative effects of APRI have been reported in many earlier literatures, these researches focused mainly on the roles played by APRI on the water saving or the crop performance, few studies have looked into the effect of APRI on the soil nutrient, particularly on the soil ${\text{NO}}_{3}^{-}$-N.

Crop yield formation is greatly influenced by the environmental factors such as light and temperature under different growth seasons (Tijskens et al., 2016). In recent years, tomato has quickly become one of the major vegetables grown in solar greenhouses of China because of its high potential yield, water productivity and profitability (Maomao et al., 2014). For greenhouse tomato, it is of great importance to select suitable transplant season. Excessive irradiance and temperature facilitate the occurrence of the blossom-end rot incidence and increase the yield loss in tomato (Kanechi et al., 2013). On the contrary, under a relatively lower temperatures (day and night temperature of 16/14°C), the early yield is delayed when compared to the conventional ones (day and night temperature of 20/18°C) although will be balanced out by higher yields in later harvests (Kläring et al., 2015).

Modern agriculture is now faced with two tasks: (1) to maintain crop yield and quality while increasing water use efficiency, and (2) to reduce agricultural pollutant outputs in greatest degree when irrigating (Djurović et al., 2016; Tang et al., 2016). In this study, it is hypothesized that the yield, quality, water saving in tomato (*Solanum lycopersicum L*) can reach a compromise under APRI. We also hypothesized that the lower water supply in APRI may keep nitrate-N in shallower soil layer, thus may increase the plant N uptake compared to conventional irrigation. Therefore, we conducted an experiment in northeastern China that using ^15^N tracing technique as research method, to investigate: (1) the effect of APRI on the tomato growth, biomass accumulation, quality, yield and WUE at different transplant time, and (2) the effect of APRI on the recovery and loss of soil ${\text{NO}}_{3}^{-}$-N. The results are expected to provide useful information for the application of APRI under greenhouse condition, and for the reutilization of soil residual fertilizer nitrogen.

## Methods

### Experiment site description

The experiments were carried out in the Production Base of Greenhouse Vegetables (longitude 126°22′E, latitude 46°12′N) of Lanxi county, Suihua city, Heilongjiang province (Experiments were permitted by the owner of the fields named Jiahui Hou). Suihua city is located in the northern hemisphere temperate zone. Suihua has four distinct seasons, with snow covering in winter season, while the climate of summer season is warm and humid. Moreover, the maximum average annual temperature from 2000 to 2013 is in a range of 18.4° to 26.6°C, while the minimum is from −13.2° to −24.8°C. The frost-free season is in a period of 120–140 days, and the sunshine duration are 2,600–2,900 h. The annual average amount of rainfall is 483 mm. The rainfalls occur intensively in summer, particularly in July and August.

**Figure 1:**
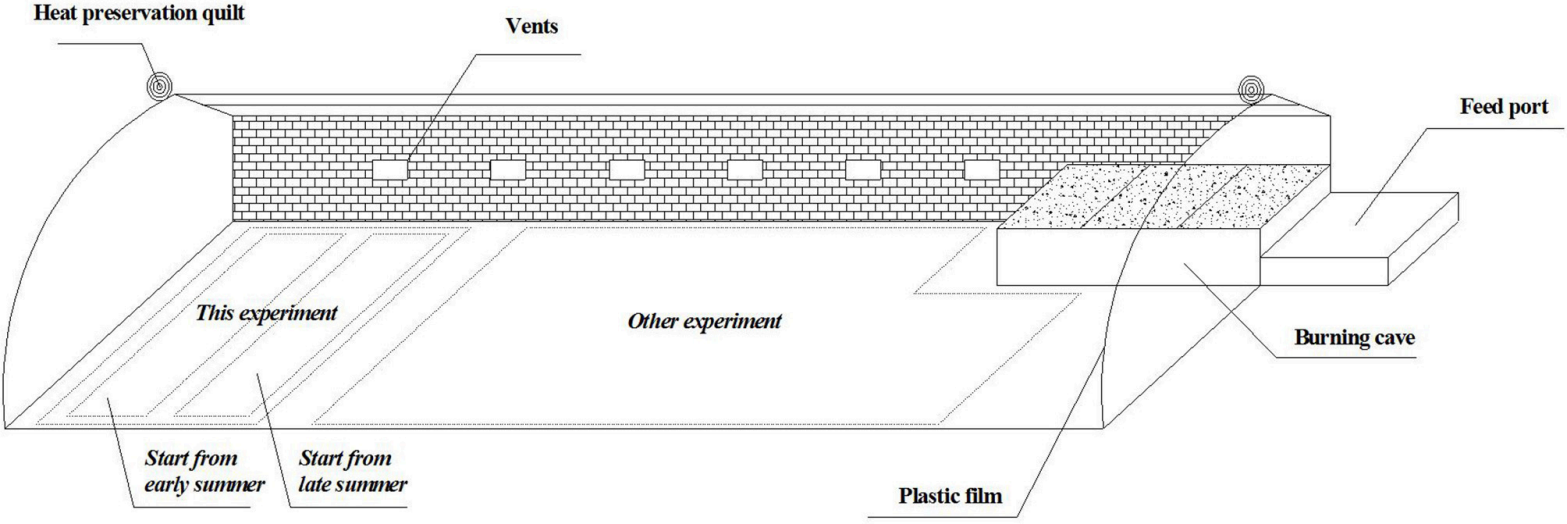
**Solar greenhouse for the experiment (During whole growth stage of crop, the day/night average temperature was 24/20°C in early summer, and 20/18°C in late summer)**.

The experiments were conducted in a solar greenhouse with span of 10 m, length of 80 m and back wall height of 3 m. Several vents were installed for ventilating and cooling when intensely high temperature occurred, and the height from the ground to the vent was 1 m (Figure 1). The crops in this study were transplanted at different dates respectively in early summer and late summer. During the whole growth stage of crop, the day/night average temperature was 24/20°C in early summer, and 20/18°C in late summer. The soil in the greenhouse was classified as silt loam, its physicochemical properties, measured prior to the early-summer experiment, were listed in Table 1.

**Table 1:** **The physical and chemical properties of the soil in the greenhouse**.

**Soil depth (cm)**	**pH**	**Bulk density (g cm^−3^)**	**Organic matter (g kg^−1^)**	**Total N (g kg^−1^)**	**Available N (mg kg^−1^)**	**Available P (mg kg^−1^)**	**Available K (mg kg^−1^)**
0–10	7.37	1.39	14.71	1.40	122.4	18.81	121.3
10–20	7.44	1.42	10.93	1.25	105.7	14.92	106.2
20–60	7.65	1.55	8.62	0.78	91.6	5.33	63.4
60–100	7.91	1.51	5.36	0.39	61.3	3.21	35.5

### Experiment design

The experiment included two irrigation patterns, two ^15^N labeled depths in soil, and two transplant dates, thus there were 2 × 2 × 2 = 8 treatments in total. The ^15^N was labeled at 10 cm and 50 cm soil depth, respectively. The irrigation patterns contained APRI and conventional irrigation (CI). The transplant dates were June 18, 2014 and August 22, 2014, respectively (differed by 9 weeks), corresponding to early and late summer. Detailed experimental design was also shown in Table 2.

**Table 2:** **Experiment designs of ^15^N labeling, irrigation methods and transplanting time**.

**Transplant time**	**Treatments**	**Irrigation patterns**	**Depths of ^15^N labeling (cm)**	**date**
Early summer	APRI10	APRI	0–20	June 18
	CI10	CI	0–20	June 18
	APRI50	APRI	40–60	June 18
	CI50	CI	40–60	June 18
Late summer	APRI10	APRI	0–20	August 22
	CI10	CI	0–20	August 22
	APRI50	APRI	40–60	August 22
	CI50	CI	40–60	August 22

*APRI represents alternate partial root-zone irrigation, and CI represents conventional irrigation*.

**Figure 2:**
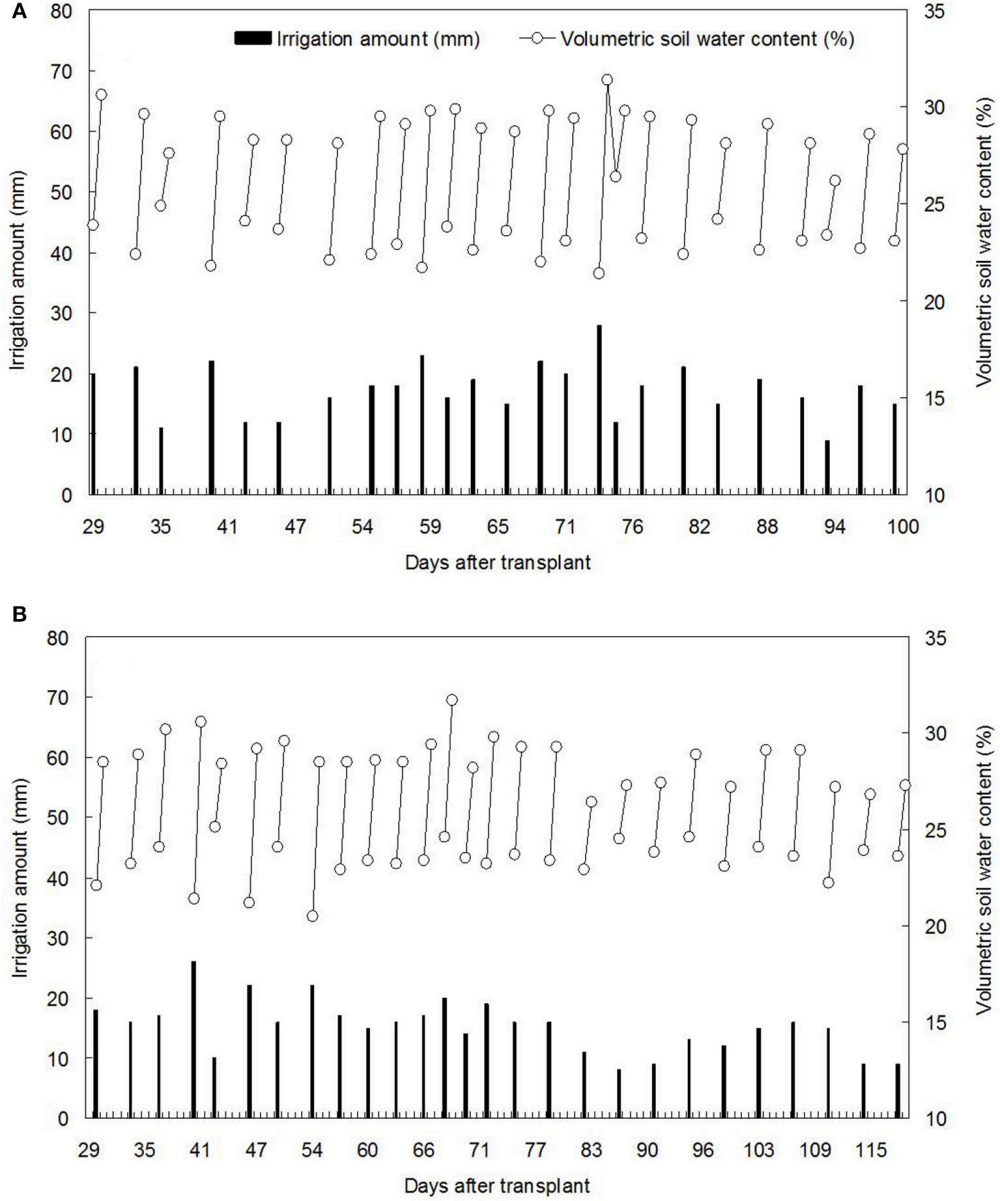
**Each irrigation and its impact on volumetric soil water content for the conventional treatments at transplant time of early summer (A)** and later summer **(B)**.

The tomato cultivar used was “*Red Ruby*.” The experiment under solar greenhouse in northern China showed that controlling the lower limit of soil moisture at 70%θ_*f*_ (field capacity in 0–20 cm soil layer, 32.6%) and upper limit at 90%θ_*f*_ could reach an optimal compromise among WUE, yield and quality of tomato (Lv, 2013). Based on the results of previous study, the soil moisture of CI in this study was controlled at a lower limit of 70%θ_*f*_, and an upper limit of 90%θ_*f*_, during the whole growth stage of tomato. Early studies proved that APRI could save 40% irrigation water while not significantly reduce the crop yield (Du et al., 2006; Wang, 2014). Thus, in our study, total irrigation amount of APRI was designed as 60% of the amount of CI. Once the soil moisture (0–20 cm layer) in CI was close to 70%θ_*f*_, the irrigation started and the amount was recorded, then 60% of the recorded irrigation amount was provided to one-side of root-zone in APRI, and next time changing to the other side. For both seasons, tomatoes were irrigated by the different patterns from 28 days after transplant (DAT), the same amount of 62 mm irrigation water among the treatments was applied for seedling survival during 0–28 DAT. The total irrigation amount of CI and APRI was recorded as 498 and 324 mm, respectively, at the transplant time of early summer (TES). Similarly, the total irrigation amount of CI and APRI was recorded as 476 and 310 mm, respectively, at the transplant time of late summer (TLS). The soil moisture was measured using the Delta-T ML2X soil moisture meter, and the irrigation was conducted by hand. Each irrigation and its impact on the volumetric soil water content for CI treatments were shown in Figure 2.

**Figure 3:**
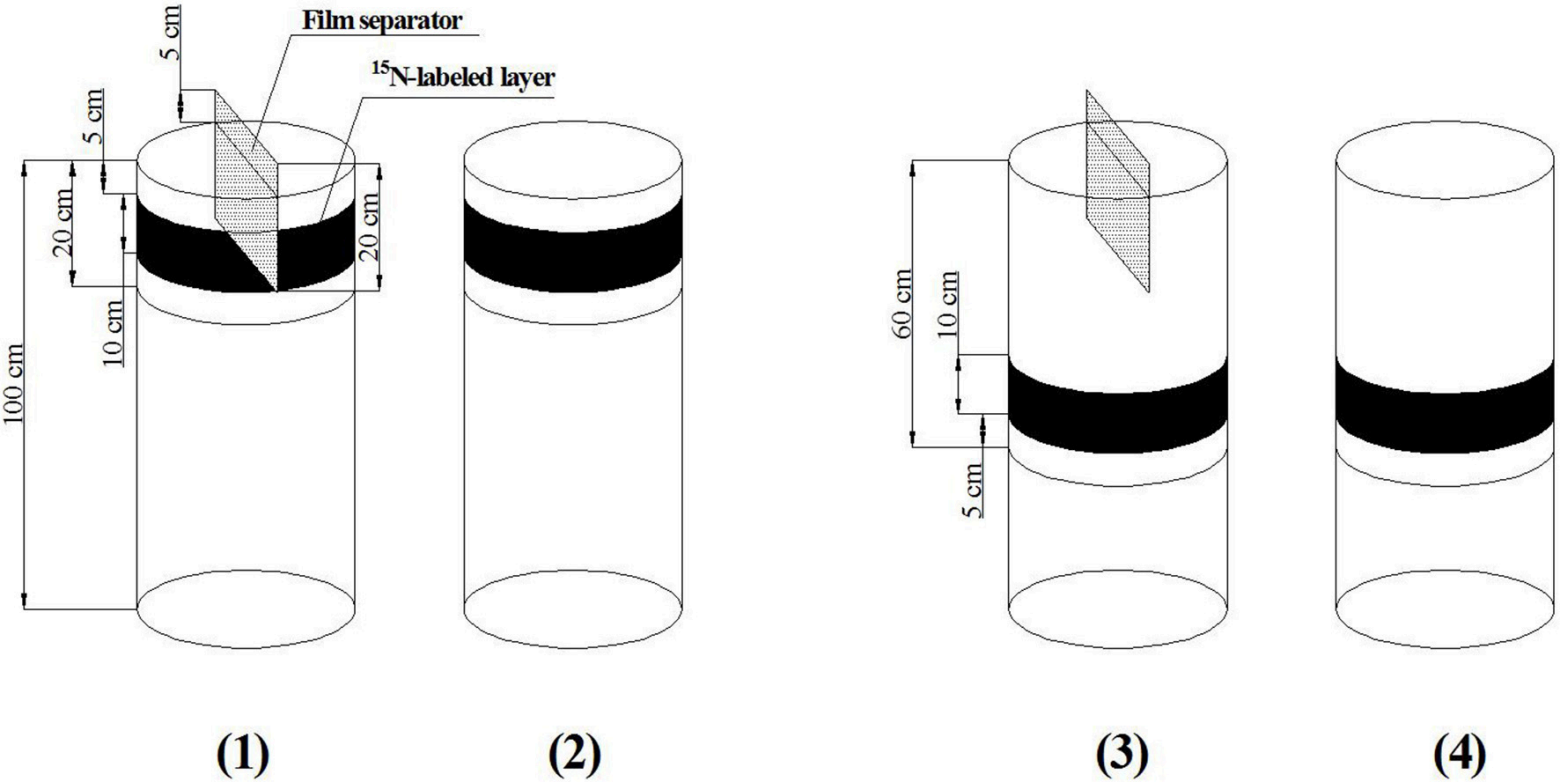
**Diagrammatic sketch of ^15^N labeling in soil column [Soil columns of (1)** and **(3)** are for the plants with alternate partial root-zone irrigation, **(2)** and **(4)** are for the plants with conventional irrigation].

The experiment was conducted in several soil columns that pre-buried in the fields. The soil column was prepared using PVC cylindrical mold with a height of 1 m and a diameter of 40 cm, and the bottom of mold was not sealed. Plastic films were used and kept closely to the inner side of the mold. The soils were digged out as 20 cm depth per layer and were filled into the mold as original layers of the field. To avoid the deflecting of mold, the backfill soils were kept the same height for inside and outside the mold during the filling process. Soils in 0–20 cm layer were mixed with NH_4_NO_3_, Ca(H_2_PO_4_)_2_ and K_2_SO_4_ to provide nutrients needed by tomato, and the dosage was 100 mg/kg N (3.5 g per column), 150 mg/kg P_2_O_5_ (5.3 g per column) and 150 mg/kg K_2_O (5.3 g per column). The K^15^NO_3_ with the abundance of 10.57% was adopted as the labeling material, and the ^15^N labeled layer was 10 cm in thickness, as was shown in Figure 2. For each soil column, the dosage of ^15^N was 466 mg. It should be noticed that, since K^15^NO_3_was used to make the ^15^N labeling, an K_2_O amount of 1.5 g was added into each column along with the ^15^N. After the soil backfilling and the ^15^N labeling, the mold was taken out from the field, leaving the plastic film separating the soils inside and outside the column. The dissolved urea was used as additional fertilizer, and was applied two times, for each time the N application amount was 60 mg/kg. The additional fertilizer was applied at 55 and 76 DAT respectively, according to local practice. Besides, a film separator with 20 cm height was buried in the middle of each soil column for APRI treatment, 5 cm height of the separator was left out of the soil surface (Figure 3). The separator opened a gap in the center for the tomato planting.

**Figure 4:**
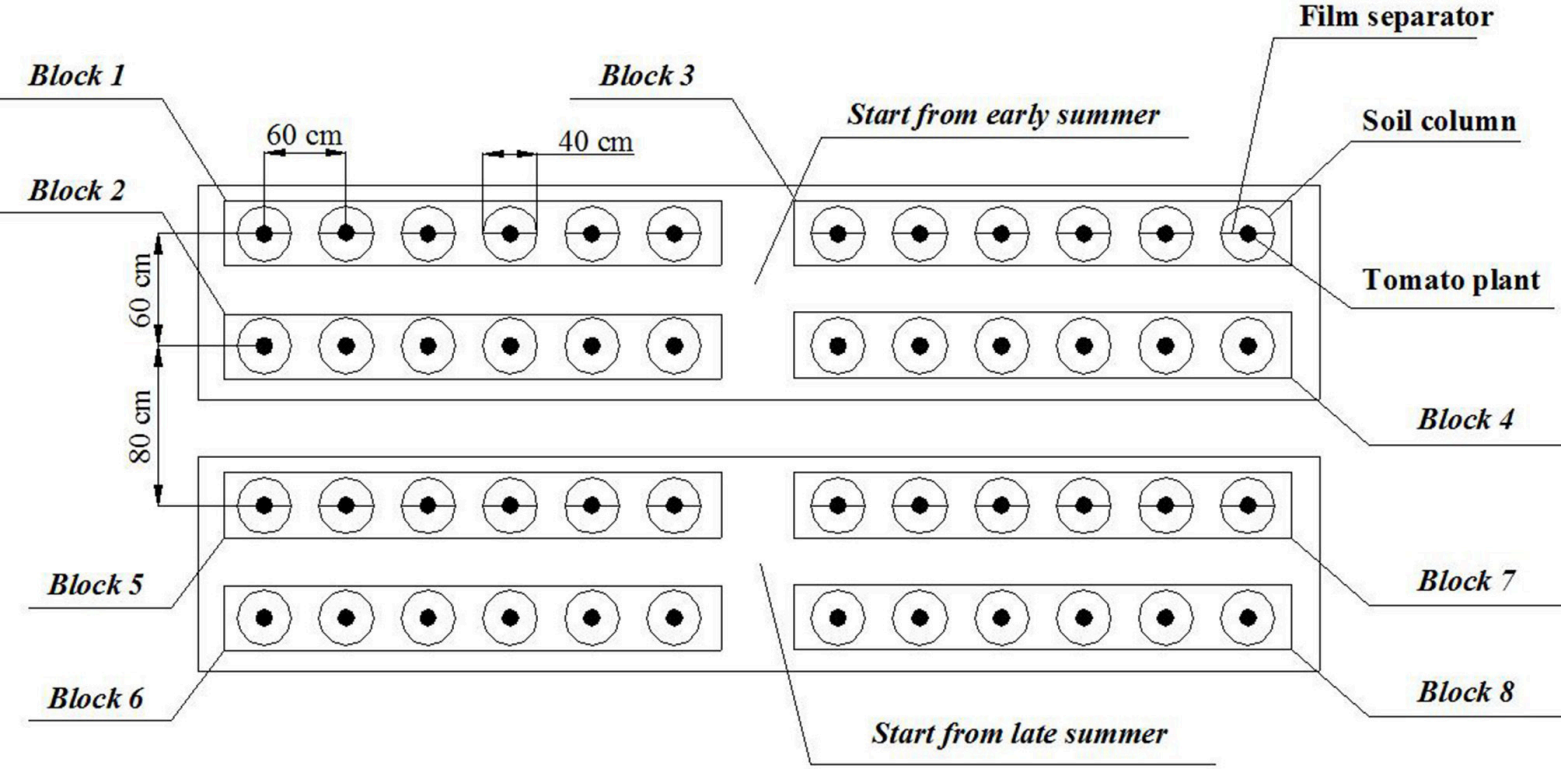
**Arrangement of soil columns (In block 1 and block 5, the plants are treated with alternate partial root-zone irrigation, and ^15^N is labeled at 10 cm depth in the soil; in block 3 and block 7, the plants are treated with alternate partial root-zone irrigation, and ^15^N is labeled at 50 cm depth in the soil; in block 2 and block 6, the plants are treated with conventional irrigation, and ^15^N is labeled at 10 cm depth in the soil; in block 4 and block 8, the plants are treated with conventional irrigation, and ^15^N is labeled at 50 cm depth in the soil)**.

To avoid the interaction in leached ^15^${\text{NO}}_{3}^{-}$ between treatments with different ^15^N labeled locations, those treatments with the same labeled location were arranged together. The detailed soil column arrangement was displayed in Figure 4. The distance between two adjacent columns was 20 cm. The distance between the two plots for different transplant time was 40 cm.

### Plant and soil sampling

The tomato fruits were harvested in batches during the maturity stage. The first harvest were done at 56–76 DAT (Table 3), and the harvest duration were from 41 to 53 days. The fallen leaves were collected during the whole growth stage of tomato. The tomato plants were collected separately as root, stem, leaf and fruit after the experiment. A 10 cm-diameter root drill (KHT-016, produced by Kanghua Electronic Instrument co., LTD, Jintan City, China) was used to collect root samples, respectively from 0–20, 20–40, 40–60, 60–80 to 80–100 cm soil layer. The root components were carefully seeked out from the soil using tweezers.

**Table 3:** **Plant Height, stem diameter and the time of first harvesting**.

**Transplant time**	**Treatments**	**30 DAT**		**60 DAT**		**First harvesting (DAT)**
		**Plant height (cm)**	**Stem diameter (cm)**	**Plant height (cm)**	**Stem diameter (cm)**	
Early summer	APRI10	53.6 ± 2.1a	0.68 ± 0.02ab	84.2 ± 4.2ab	1.19 ± 0.03a	61
	CI10	55.8 ± 3.4a	0.72 ± 0.03a	90.3 ± 2.1a	1.22 ± 0.03a	65
	APRI50	45.9 ± 1.9bc	0.59 ± 0.01d	79.2 ± 3.3bc	1.05 ± 0.06b	56
	CI50	43.5 ± 2.6c	0.63 ± 0.02c	80.6 ± 2.6bc	1.08 ± 0.05b	61
Late summer	APRI10	48.1 ± 1.4b	0.65 ± 0.02bc	78.6 ± 0.8c	1.03 ± 0.02b	73
	CI10	48.9 ± 2.2b	0.65 ± 0.02bc	83.1 ± 3.3b	1.07 ± 0.03b	76
	APRI50	39.2 ± 0.6d	0.59 ± 0.01d	74.3 ± 1.4d	0.94 ± 0.02c	70
	CI50	40.1 ± 2.8cd	0.62 ± 0.02cd	75.9 ± 3.2cd	0.96 ± 0.04bc	71
Transplant time (TT)		^**^	^*^	^**^	^**^	—
^15^N labeling (NL)		^**^	^**^	^**^	^**^	—
Irrigation pattern (IP)		ns	ns	ns	ns	—
TT × NL		ns	ns	ns	ns	—
TT × IP		ns	ns	ns	ns	—
NL × IP		ns	ns	ns	ns	—
TT × NL × IP		ns	ns	ns	ns	—

*APRI10 and APRI50 represent that ^15^N is labeled at 10 and 50 cm soil depths respectively under alternate partial root-zone irrigation, CI10 and CI50 represent that ^15^N is labeled at 10 and 50 cm soil depths respectively under conventional irrigation. DAT represents days after transplanted. In the same column, the means followed by the same letter (a, b, c, d) do not differ significantly at 5% level, according to Duncan's multiple range test. Each value is the mean ± SD. According to local experience, 30 and 60 DAT correspond approximately to flowering and fruit-set stage of tomato, respectively*.

*, ** *and ns indicate that the experimental treatment has a significant (at 0.05 level) effect, an extremely significant (at 0.01 level) effect, and no significant effect on the plant growth indicator, respectively*.

After harvest, soil samples were collected using a soil auger as 10 cm per layer. Ten samples in a total depth of 1 m were collected for each soil column.

### Analytical methods

At each harvest time, the number and weight of tomatoes were recorded, and the tomato yield was calculated after the last harvest.

The tomatoes in the first and third layer of the plant were used for the quality determination. In each treatment, twelve ripening (when the fruits turned red) fruits (6 from first layer and 6 from the third) with similar appearance were randomly collected from the six plants. For each fruit, 10 g tomato flesh was taken along the longitudinal axis and homogenized for quality measurements. The following components contributed greatly to the tomato quality: volume, density, soluble solids, total acid, vitamin C and sugar/acid ratio. The fruit volume was measured by the displacement method. The soluble solids were measured using a ACT-1E digital refractometer (ATAGO company, Japan). The total sugar was measured by Fehling reagent titration method. The total acid was measured by the sodium hydroxide titration method. The vitamin C content was measured by 2, 6-dichloroindophenol titrimetric method (Zhai et al., 2015).

The plant samples were placed into an oven and were dried firstly at 105°C for 30 min, then at 70°C until achieving the constant weight. The soil samples were air dried naturally in open space. Dried soil samples were ground and sieved through 0.15 mm mesh for ^15^N measuring. The ^15^N atom percent excess in plant and soil samples was measured using the mass spectrometer (Finniga-Mat-251, Finnigan, Germany) at Nanjing Institute of Soil Science, CAS.

The root samples of tomato were cleaned and scanned by the EPSON EXPRESSION 1680 scanner, then analyzed using the WinRHIZO software to get the data of root length density.

### Calculations and statistical analysis

Tomato LAI was simulated using the model proposed by Qin et al. (2008):

(1) $$\begin{array}{l} \text{LAI}=\text{LA}{\text{I}}_{\text{M}}{\left(1+\left(1\text{-}\beta \right){\text{e}}^{\text{-}\alpha \left(\text{t-}\tau \right)}\right)}^{\text{-}1}+\text{LA}{\text{I}}_{\text{0}}\left(1\text{-}\beta \right) \end{array}$$

Where, LAI and t is leaf area index and the days after transplanted, respectively. LAI_M_, LAI_0_, α, β, and τ are the parameters that will be determined according to the measured values of LAI and t (LAI were measured during 10–90 DAT, in 10-day intervals). LAI_M_ is the theoretical upper limit of LAI, LAI_0_ is the theoretical lower limit of LAI, α, β is the constants, τ is the days that needed to reach 1/2 LAI_M_.

The WUE (kg m^−3^) is calculated by the following equation:

(2) $$\begin{array}{l} \text{WUE}=\text{Y/ET} \end{array}$$

where Y is the tomato yield (t ha^−1^), ET is the evapotranspiration (mm). ET is calculated using the water balance equation of the farm land (Hou and Shao, 2016):

(3) $$\begin{array}{l} \text{ET}=\text{P}+\text{I}+\text{U-R-D-}\Delta \text{W} \end{array}$$

where, P is the valid rainfall (mm), I is the irrigation amount (mm), U is the groundwater recharge (mm), R is the runoff (mm), D is the deep percolation (mm), and ΔW is the variation of soil moisture before and after the experiment (mm). For this study, P, U (the ground water level is below 9 m), R and D can be ignored. The equation can thus be simplified as:

(4) $$\begin{array}{l} \text{ET}=\text{I-}\Delta \text{W} \end{array}$$

The ^15^N use efficiency (^15^NUE, %) was calculated as (Liang et al., 2013):

(5) $$\begin{array}{lll} N\text{dff} & = & {\text{C}}_{\text{s}}\times \frac{{\text{E}}_{\text{s}}}{{\text{E}}_{\text{f}}} \end{array}$$

(6) N15UE=(NdffMf)×100

Where, *N*dff is the ^15^N amount in tomato organ (mg), C_s_ is the total N amount in tomato organ (mg), E_s_ is the ^15^N atom percentage excess of tomato organ, E_f_ is the ^15^N atom percentage excess of the ^15^N labeled fertilizer, *M*_f_ is the amount of ^15^N labeled fertilizer (mg).

The amount of^15^N recovery amount (mg per soil column) contains the ^15^N in 0–100 cm soil layer and the ^15^N absorbed by tomato plant. The^15^N recovery rate is the ratio of ^15^N recovery amount and ^15^N application amount (Liang et al., 2013).

The ^15^N loss (mg per soil column) is calculated using the ^15^N application amount minus the ^15^N recovery amount. The ^15^N loss rate is the ratio of ^15^N loss and ^15^N application amount (Hou et al., 2017).

The data were compared statistically using Duncan's multiple range test in SPSS software Version 17.0. Data from the same treatment but different planting seasons were also statistically analyzed.

## Result and discussion

### Crop performance

As was shown in Table 3, both plant height and stem diameter were significantly affected by ^15^N labeled depth (*p* < 0.01) or transplant time (*p* < 0.05), but were not significantly (*p* > 0.05) affected by irrigation pattern. The insignificant difference of plant growth between two irrigation patterns might be that: (1) APRI reduced the ineffective water consumption for crop (Centritto et al., 2005), thus the growth of tomato have not been obviously affected. (2) APRI was proved by the early study to maintain the optimal aeration and moisture condition in soil and enhance the activities of soil microorganisms (Wang, 2008), which might have benefited the plant N uptake and the plant growth, although the irrigation amount in APRI was lower. At 30 DAT, compared to the labeled depth of 50 cm, 10 cm labeled depth significantly (*p* < 0.05) increased the plant height by 16.8–28.3%, and the stem diameter by 4.8–15.3%. At 60 DAT, the plant height, as well as stem diameter, showed no significant (*p* > 0.05) difference between APRI and CI treatments, while they were significantly (*p* < 0.05) increased by the shallower labeled depth of ^15^N. CI10 registered greatest plant height (90.3 cm) and stem diameter (1.22 cm) at TES, while the lowest (74.3 cm and 0.94 cm) were in APRI50 at TLS. Generally, the tomatoes with TES obtained higher plant height and stem diameter than that with TLS, when the irrigation pattern and labeled depth were the same. Compared to CI, APRI delayed the time of first harvesting, which was particularly obvious at TES. Moreover, the duration needed for the first harvesting was noticeably influenced by the transplant time. TLS delayed 11.8 days, on average, in the first harvest of tomato, when compared to TES. Similar result obtained by Kläring (Kläring et al., 2015) showed that one Kelvin reduction in temperature would result in a 3.5-day delay of the first harvest.

**Figure 5:**
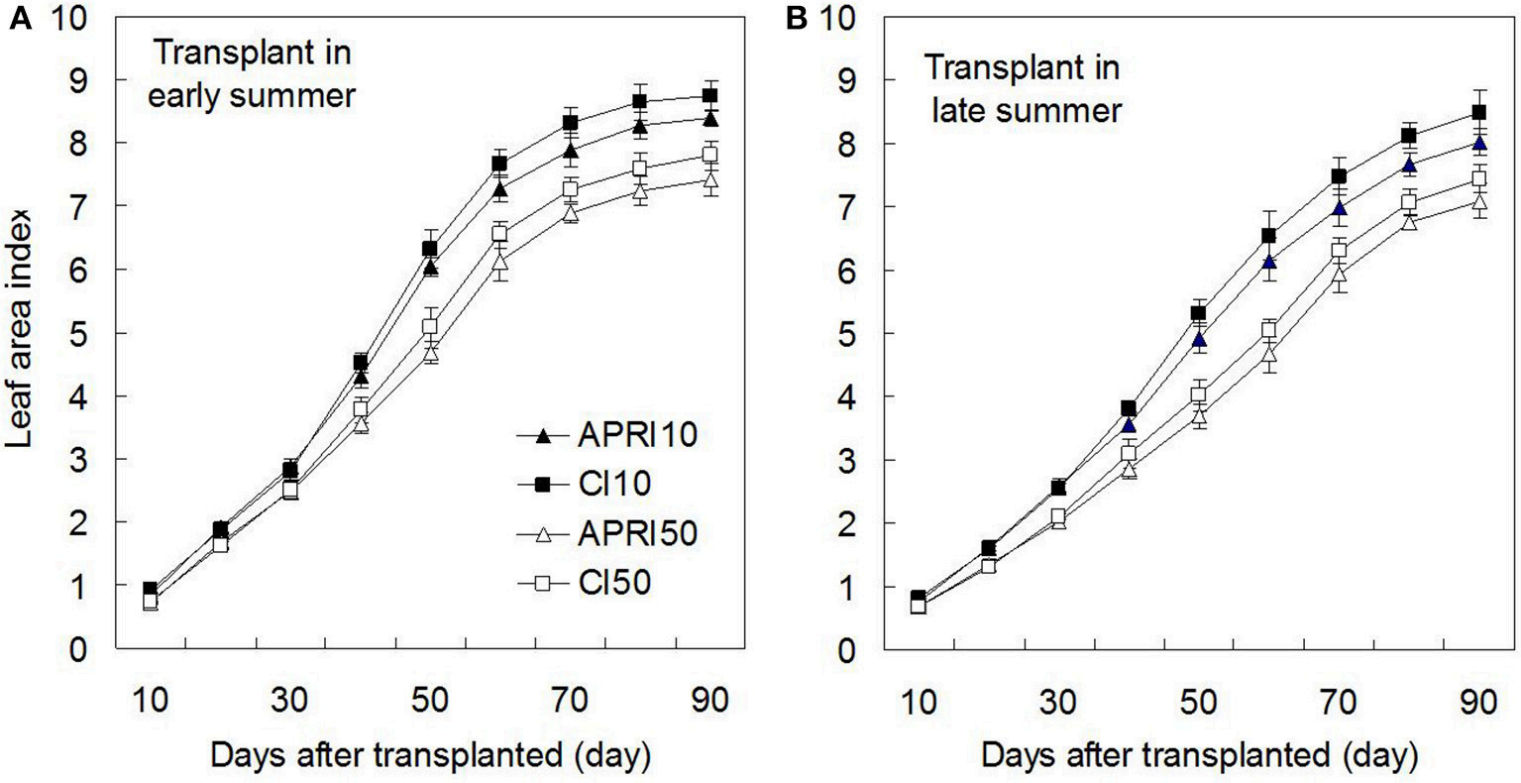
**Tomato leaf area index varying with the days after transplanted (APRI10 and APRI50 represent that ^15^N is labeled at 10 and 50 cm soil depths respectively under alternate partial root-zone irrigation, while CI10 and CI50 represent that ^15^N is labeled at 10 and 50 cm soil depths respectively under conventional irrigation)**. Tomato leaf area index varying with the days after transplanted at different transplant times of early summer **(A)** and late summer **(B)**.

The response of LAI to the treatments varying with DAT is shown in Figure 5. Under the same DAT, the labeled depth of 10 cm significantly (*p* < 0.05) increased the LAI compared to that of 50 cm. Nevertheless, no obvious difference of LAI was found between APRI and CI except the slight decrease found in APRI. There were differences between TES and TLS in the dynamics of LAI. Compared to TES, TLS achieved a greater increase rate of LAI after 60 DAT, although LAI at TLS was smaller than that at TES at any time point during 0–60 DAT. At 90 DAT, the greatest LAI were found in CI10, and were 8.8 and 8.5 for TES and TLS, respectively.

The simulation model well reflected the dynamic of LAI (Table 4), with correlation coefficient of 0.978–0.999. Compared to CI, APRI reduced the time needed by the tomato to obtain 1/2 LAI_M_, this was more obvious at TES. As was calculated by the model, APRI slightly decrease the LAI_M_ by 2.3–3.8%. However, LAI_M_ was noticeably increased by the shallower labeled depth of ^15^N, 10 cm labeled depth increased which by 12.0–14.3% and 13.0–13.2%, respectively under APRI and CI. The model also showed that under the same irrigation pattern and labeled depth of ^15^N, LAI_M_ showed almost no difference between TES and TLS.

**Table 4:** **The parameters and correlation coefficient of the LAI simulation model**.

**Transplant time**	**Treatments**	**LAI_0_**	**LAI_M_**	**α**	**β**	**τ**	**Correlation coefficient**
Early summer	APRI10	0.191	8.64	0.074	0.504	49.1	0.997
	CI10	1.086	8.84	0.081	0.863	64.3	0.997
	APRI50	0.062	7.56	0.069	−0.217	38.5	0.993
	CI50	0.220	7.82	0.075	0.128	43.7	0.997
Late summer	APRI10	0.011	8.51	0.061	0.464	54.8	0.998
	CI10	0.053	8.83	0.066	0.460	53.4	0.999
	APRI50	−0.240	7.60	0.057	−0.028	45.1	0.978
	CI50	−0.329	7.80	0.061	0.098	45.4	0.979

*The tomato LAI was simulated by the model LAI = LAI_M_[1+(1-β)e^−α(t−τ)^]^−1^+LAI_0_(1-β). Where, LAI and t is the leaf area index and the days after transplanted, respectively. LAI_M_, LAI_0_, α, β and τ are the parameters that will be determined according to the measured values of LAI and t. LAI_M_ is the theoretical upper limit of LAI, LAI_0_ is the theoretical lower limit of LAI, α, β is the constants, τ is the days that needed to reach 1/2 LAI_M_*.

### Biomass

For both transplant times, APRI had no significant (*p* > 0.05) effect on the biomass of leaf, stem, fruit, as well as their sum, when the labeled depths were the same. While under the same irrigation patterns, the biomass of the plant parts were significantly (*p* < 0.01) influenced by the labeled depth of ^15^N (Table 5). Compared to 50 cm, the 10 cm labeled depth significantly (*p* < 0.05) increased the leaf biomass by 11.3–12.6%, the stem by 11.2–24.2% and the fruit by 9.5–13.0%, for both transplant times. The applied ^15^${\text{NO}}_{3}^{-}$ could have contributed to a higher dry matter accumulation of tomato (Wang, 2014; Badr et al., 2016). An insignificant decrease in biomass of leaf, stem and fruit was found in TLS when compared to TES. The tomato with CI10 at TES obtained highest fruit biomass of 249.8 g plant^−1^, although it had not significant (*p* > 0.05) difference compared to that with APRI10. The total biomass aboveground were ranged from 458.1 to 539.1 g plant^−1^, the highest was registered by CI10 at TES while the lowest was in APRI50 at TLS.

**Table 5:** **Biomass of the tomato aboveground part**.

**Transplant time**	**Treatments**	**Leaf (g plant^−1^)**	**Stem (g plant^−1^)**	**Fruit (g plant^−1^)**	**Total (g plant^−1^)**
Early summer	APRI10	175.7 ± 7.3a	108.7 ± 5.2a	247.4 ± 7.5a	531.8 ± 20.0a
	CI10	177.2 ± 8.2a	112.2 ± 8.7a	249.8 ± 10.6a	539.1 ± 27.3a
	APRI50	156.1 ± 9.5cd	89.4 ± 3.6c	220.1 ± 16.0bc	465.7 ± 28.9c
	CI50	159.2 ± 6.8bcd	93.2 ± 5.2bc	222.2 ± 10.6bc	474.6 ± 22.2bc
Late summer	APRI10	172.2 ± 8.1abc	103.4 ± 6.0ab	243.4 ± 8.7ab	519.0 ± 22.4ab
	CI10	174.4 ± 8.1ab	104.2 ± 5.6ab	240.8 ± 11.2abc	519.3 ± 24.0ab
	APRI50	152.1 ± 8.3d	88.6 ± 4.1c	217.5 ± 4.1c	458.1 ± 16.4c
	CI50	155.0 ± 2.6cd	90.3 ± 5.0c	219.0 ± 11.4bc	464.3 ± 17.3c
Transplant time (TT)		ns	ns	ns	ns
^15^N labeling (NL)		^**^	^**^	^**^	^**^
Irrigation pattern (IP)		ns	ns	ns	ns
TT × NL		ns	ns	ns	ns
TT × IP		ns	ns	ns	ns
NL × IP		ns	ns	ns	ns
TT × NL × IP		ns	ns	ns	ns

*APRI10 and APRI50 represent that ^15^N is labeled at 10 and 50 cm soil depths respectively under alternate partial root-zone irrigation, CI10 and CI50 represent that ^15^N is labeled at 10 and 50 cm soil depths respectively under conventional irrigation. In the same column, the means followed by the same letter (a, b, c, d) do not differ significantly at 5% level, according to Duncan's multiple range test. Each value is the mean ± SD*.

** *and ns indicate that the treatment has an extremely significant (at 0.01 level) effect, and no significant effect on the biomass, respectively*.

### Root attributes

Overall, the root dry weight and length density at all soil layers were significantly affected by the irrigation pattern (Table 6). Compared to CI, APRI promoted the root growth in varying degrees, agreeing with Wang et al. (2012) findings that the crop root was enlarged by APRI compared to full irrigation. This mainly due to the fact that APRI distributed more photosynthetic products to the root, moreover, the dry-wet condition under APRI stimulated the compensatory growth for root (Li et al., 2011). Oppositely, Abrisqueta et al. (2008) reported a 42% reduction in root length density under APRI, possibly because he adopted a lower irrigation amount (50% relative to the full irrigated treatment). Besides, the root attributes were also significantly influenced by ^15^N labeling, this influence was more evident in the labeling layer, proving that the tomato root was sensitive to nitrate ^15^N supply. Early study also showed that ${\text{NO}}_{3}^{-}$-N could induce the growth and development of crop lateral roots effectively, thus increase root dry weight and length density (Centritto et al., 2005). In this study, the greatest root dry weight and length density were both found in APRI10 at TES, and were 0.352 mg cm^−3^ and 0.672 cm cm^−3^, respectively. Although, the transplant time had no obvious impact on root dry weight, as well as root length density, both of them were found slightly lower at TLS compared to that at TES.

**Table 6:** **Dry weight and length density of tomato root under different treatments**.

**Transplant time**	**Treatments**	**Root dry weight (mg cm^−3^)**					**Root length density (cm cm^−3^)**				
		**0–20 cm**	**20–40 cm**	**40–60 cm**	**60–80 cm**	**80–100 cm**	**0–20 cm**	**20–40 cm**	**40–60 cm**	**60–80 cm**	**80–100 cm**
Early summer	APRI10	0.352 ± 0.016a	0.050 ± 0.002a	0.035 ± 0.002d	0.028 ± 0.002ab	0.009 ± 0.001b	0.672 ± 0.033a	0.340 ± 0.013a	0.240 ± 0.008c	0.131 ± 0.007b	0.051 ± 0.002a
	CI10	0.285 ± 0.009b	0.048 ± 0.002ab	0.028 ± 0.001ef	0.024 ± 0.001bc	0.009 ± 0.002b	0.608 ± 0.024bcd	0.262 ± 0.007c	0.212 ± 0.009cd	0.121 ± 0.007bc	0.050 ± 0.001a
	APRI50	0.304 ± 0.018b	0.049 ± 0.001ab	0.051 ± 0.002ab	0.030 ± 0.002a	0.013 ± 0.001a	0.621 ± 0.023abc	0.310 ± 0.008b	0.456 ± 0.025a	0.155 ± 0.009a	0.053 ± 0.002a
	CI50	0.235 ± 0.015c	0.043 ± 0.002c	0.047 ± 0.002bc	0.026 ± 0.002bc	0.011 ± 0.001ab	0.582 ± 0.033cd	0.249 ± 0.013c	0.398 ± 0.014b	0.146 ± 0.007a	0.052 ± 0.001a
Late summer	APRI10	0.311 ± 0.016b	0.048 ± 0.001ab	0.031 ± 0.002de	0.026 ± 0.001abc	0.010 ± 0.002ab	0.652 ± 0.026ab	0.336 ± 0.007a	0.227 ± 0.006cd	0.121 ± 0.007bc	0.051 ± 0.002a
	CI10	0.285 ± 0.011b	0.045 ± 0.001bc	0.026 ± 0.002f	0.022 ± 0.002cd	0.008 ± 0.001b	0.591 ± 0.016cd	0.289 ± 0.004b	0.203 ± 0.007d	0.115 ± 0.003c	0.051 ± 0.001a
	APRI50	0.283 ± 0.007b	0.046 ± 0.003bc	0.053 ± 0.002a	0.024 ± 0.002bc	0.011 ± 0.001ab	0.634 ± 0.026abc	0.298 ± 0.020b	0.410 ± 0.009b	0.158 ± 0.003a	0.053 ± 0.001a
	CI50	0.254 ± 0.009c	0.044 ± 0.002c	0.045 ± 0.002c	0.019 ± 0.002d	0.008 ± 0.001b	0.552 ± 0.014d	0.261 ± 0.016c	0.383 ± 0.012b	0.151 ± 0.003a	0.053 ± 0.001a
Transplant time (TT)		ns	ns	ns	^**^	ns	ns	ns	^**^	ns	ns
^15^N labeling (NL)		^**^	^**^	^**^	ns	^*^	^*^	^**^	^**^	^**^	^*^
Irrigation pattern (IP)		^**^	^**^	^**^	^**^	^*^	^**^	^**^	^**^	^*^	ns
TT × NL		ns	ns	ns	^*^	ns	ns	ns	ns	ns	ns
TT × IP		^**^	ns	ns	ns	ns	ns	^*^	ns	ns	ns
NL × IP		ns	ns	ns	ns	ns	ns	ns	ns	ns	ns
TT × NL × IP		ns	ns	ns	ns	ns	ns	ns	ns	ns	ns

*APRI10 and APRI50 represent that ^15^N is labeled at 10 and 50 cm soil depths respectively under alternate partial root-zone irrigation, CI10 and CI50 represent that ^15^N is labeled at 10 and 50 cm soil depths respectively under conventional irrigation. “0–20 cm,” “20–40 cm,” “40–60 cm,” “60–80 cm,” and “80–100 cm” represent the locations in soil profile. In the same column, the means followed by the same letter (a, b, c, d, e, f) do not differ significantly at 5% level, according to Duncan's multiple range test. Each value is the mean ± SD*.

*, ** *and ns indicate that the experimental treatment has a significant (at 0.05 level) effect, an extremely significant (at 0.01 level) effect, and no significant effect on the root indicator, respectively*.

### Yield and WUE

**Figure 6:**
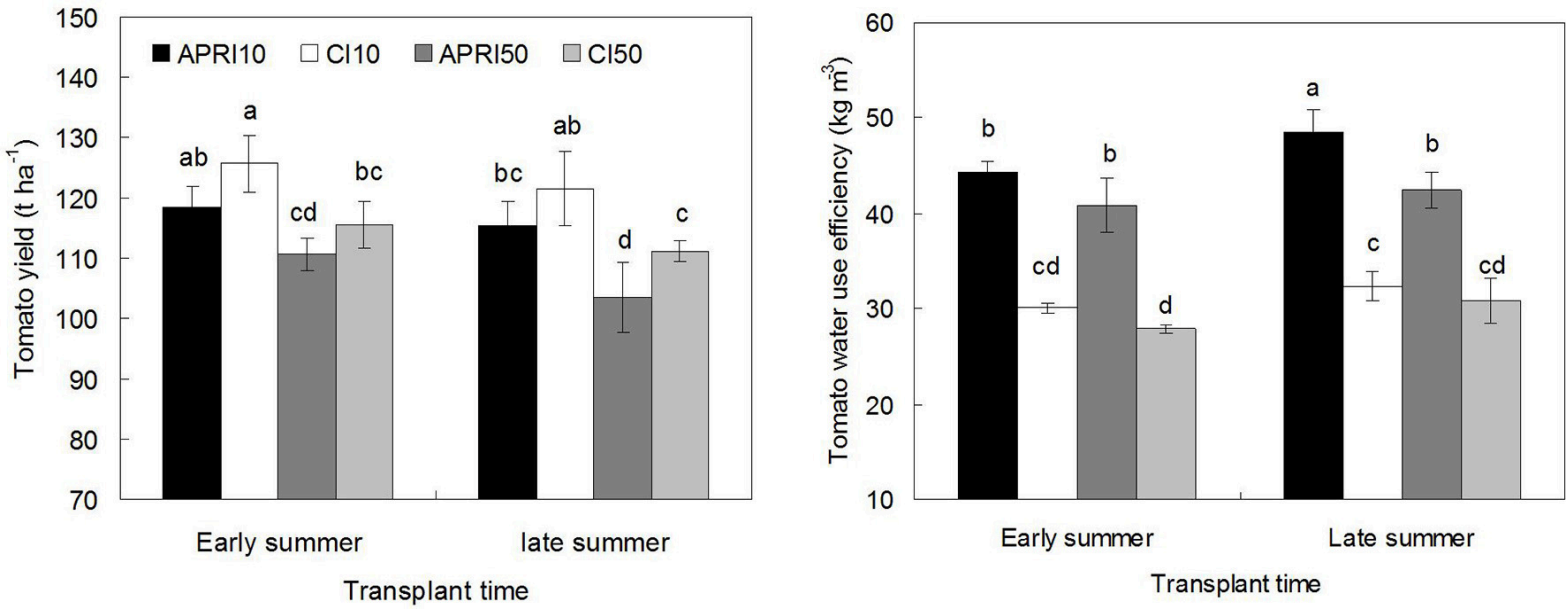
**Yield and water use efficiency of tomato with different treatments (APRI10 and APRI50 represent that ^15^N is labeled at 10 and 50 cm soil depths respectively under alternate partial root-zone irrigation, CI10, and CI50 represent that ^15^N is labeled at 10 and 50 cm soil depths respectively under conventional irrigation**. The means followed by the same letter (a, b, c, d) do not differ significantly at 5% level according to Duncan's multiple range test, and the eight means were compared together. Each value is the mean ± SD).

Compared to CI, APRI slightly decreased the yield of tomato (Figure 6). APRI10 reduced tomato yield by 6.1 and 5.4%, and APRI50 reduced it by 4.4 and 7.4%, respectively corresponding to TES and TLS, but the yield reduction by APRI was not significant (*p* > 0.05). The ^15^N labeled depth had obvious effects on the yield of tomato. APRI10 significantly (*p* < 0.05) increased the yield by 7.0 and 11.4%, respectively at TES and TLS, when compared to APRI50. Similarly, CI10 significantly (*p* < 0.05) increased the yield by 8.7 and 9.3% compared to CI50. With regards to the significant difference in yield and growth of the two ^15^N labeled depth treatments, it could be explained by that, when ^15^N was at 10 cm layer, equivalent to 13.3% higher amount of basal N was applied for the early growth of plant; while if at 50 cm layer, when the tomato root was able to capture ^15^N, great amount of the ^15^N have been leached below the main root zone and remained unavailable for utilization by the tomato root. In addition, although TES increased the tomato yield relative to TLS, the yield increase by TES was not significant (*p* > 0.05).

In comparison to CI, the APRI treatments significantly (*p* < 0.05) increased tomato WUE by 37.6–49.9%. However, the WUE was basically unaffected by the ^15^N labeled depth. Besides, the WUE of tomato at TES were slightly higher relative to that at TLS, while the difference of WUE between TES and TLS was not significant (*p* > 0.05) except that found in APRI10.

To find the compromise between yield producing and water saving, is the key task for developing an optimal irrigation scheme (Wang et al., 2011). The insignificant decrease in yield but significant increase in WUE by this study confirmed the previous findings by Kirda et al. (2004). Accumulated results on that APRI allowed considerable water savings while maintained yield were not only found in tomato (Wei et al., 2016), but in many other crops such as potato (Sun et al., 2013), maize (Wang et al., 2012), apple (Du et al., 2017), grape (Du et al., 2008), peach (Abrisqueta et al., 2008), and so on.

### Fruit quality

The indicators that contributed greatly to fruit quality were shown in Table 7 for the treatments. Compared to CI, APRI had no significant effect (*p* > 0.05) on the fruit density and volume, while it increased the contents of soluble solid, total acid, vitamin C and sugar/acid ratio in various degrees. The highest soluble solid, vitamin C contents and sugar/acid ratio were all found in APRI10 at TLS, and were 6.3%, 14.2 mg 100g^−1^ and 10.2, respectively. This indicated that APRI contributed to a higher nutrient accumulation and a better taste of fruit compared to CI. Our result confirmed the result of Yang (Yang et al., 2012) who reported that APRI increased contents of vitamin C and soluble sugar in tomato by 12.6 and 4.5% in comparison to CI. Zegbe (Zegbe et al., 2004) noted that APRI could advance the maturity of tomato fruit early, make fruit redder, increase the soluble solid content, and promote the sugar transfer from the vegetative organs to fruits to ensure fruit development thus improve the fruit quality. Similar research by Du (Du et al., 2008) showed that APRI increased vitamin C content in the fruit of grape by 15.3–42.2% and the ratio of total soluble solids/titrated acid as compared to conventional drip irrigation. The previous study revealed that when reducing irrigation water supply, the water consumption used for osmotic regulation in peel was reduced, leading to an increase in vitamin C; meanwhile, the content of sugar that entering from phloem to fruit was increased, this contributed to a higher soluble solid content in tomato (Mahajan and Singh, 2006).

**Table 7:** **Tomato quality indicators under different treatments**.

**Transplant time**	**Treatments**	**Density (g cm^−3^)**	**Volume (cm^3^)**	**Soluble solid (%)**	**Total acid (g 100g^−1^)**	**Vitamin C (mg 100g^−1^)**	**Sugar/acid ratio**
Early summer	APRI10	0.942 ± 0.013a	122.6 ± 3.7c	6.2 ± 0.5ab	0.60 ± 0.05ab	13.1 ± 0.6ab	9.8 ± 0.3a
	CI10	0.933 ± 0.012a	124.3 ± 3.2c	5.1 ± 0.3cd	0.54 ± 0.04bc	12.2 ± 0.4b	9.2 ± 0.2b
	APRI50	0.938 ± 0.007a	118.4 ± 5.2c	5.3 ± 0.2cd	0.59 ± 0.04ab	11.2 ± 0.5c	8.8 ± 0.4bc
	CI50	0.936 ± 0.008a	122.1 ± 4.1c	4.7 ± 0.4d	0.49 ± 0.02c	10.9 ± 0.4c	8.5 ± 0.2c
Late summer	APRI10	0.950 ± 0.011a	135.5 ± 5.2ab	6.3 ± 0.3a	0.63 ± 0.04a	14.2 ± 1.1a	10.2 ± 0.5a
	CI10	0.933 ± 0.015a	142.6 ± 3.7a	5.5 ± 0.4bc	0.57 ± 0.05ab	12.6 ± 0.2b	9.4 ± 0.4ab
	APRI50	0.948 ± 0.009a	133.8 ± 3.4b	5.8 ± 0.3ab	0.57 ± 0.05ab	12.7 ± 0.9ab	8.9 ± 0.3bc
	CI50	0.935 ± 0.010a	133.3 ± 2.2b	5.0 ± 0.2cd	0.52 ± 0.03bc	11.4 ± 0.3c	8.2 ± 0.5c
Transplant time (TT)		ns	^**^	^*^	ns	ns	ns
^15^N labeling (NL)		ns	ns	^*^	ns	^**^	^**^
Irrigation pattern (IP)		ns	ns	^**^	^*^	^**^	^*^
TT × NL		ns	^*^	ns	ns	ns	ns
TT × IP		ns	ns	ns	ns	ns	ns
NL × IP		ns	ns	ns	ns	ns	ns
TT × NL × IP		ns	ns	ns	ns	ns	ns

*APRI10 and APRI50 represent that ^15^N is labeled at 10 and 50 cm soil depths respectively under alternate partial root-zone irrigation, CI10 and CI50 represent that ^15^N is labeled at 10 and 50 cm soil depths respectively under conventional irrigation. In the same column, the means followed by the same letter (a, b, c, d) do not differ significantly at 5% level, according to Duncan's multiple range test. Each value is the mean ± SD*.

*, ** *and ns indicate that the experimental treatment has a significant (at 0.05 level) effect, an extremely significant (at 0.01 level) effect, and no significant effect on the quality indicator, respectively*.

Otherwise, although the density, volume, contents of soluble solid and total acid were not obviously influenced by the ^15^N labeled depth, the vitamin C content and sugar/acid ratio were significantly (*p* < 0.05) increased by the labeled depth of 10 cm. Compared to APRI50, APRI10 increased the vitamin C content and sugar/acid ratio by 17.0 and 11.4% at TES, and 11.8 and 14.6% at TLS. Similarly, compared to CI50, CI10 increased the vitamin C content and sugar/acid ratio by 11.9 and 8.2% at TES, and 10.5 and 14.6% at TLS.

Overall, the transplant time had no significant (*p* > 0.05) effect on the soluble solid, total acid, vitamin C contents and sugar/acid ratio. However, the volume of tomato at TLS were significantly (*p* < 0.05) greater than that at TES, when the irrigation pattern and the labeled depth were the same, this has proved the Kläring's (Kläring et al., 2015) findings. The greatest tomato volume (142.6 cm^3^) was registered by CI10 at TLS, while the lowest (118.4 cm^3^) was in APRI50 at TES.

### ^15^N uptake

Controlling nitrate outputs from arable land has become an arduous task for modern agriculture in China (Ju et al., 2006; Lenka et al., 2013; Wang Z. H. et al., 2015). Taking into account the likely contribution of the water-saving irrigation to crop nitrate utilization, is an alternative opinion for increasing nitrate nitrogen recovery. In this study, the ^15^N uptake in all organs were significantly (*p* < 0.05) affected by irrigation pattern (Table 8). Overall, when the ^15^N was labeled at 50 cm soil depth, the ^15^N amount in each plant part under APRI was significantly (*p* < 0.05) higher than that under CI. In comparison to CI50, APRI50 significantly (*p* < 0.05) increased the ^15^N amount of the whole plant by 13.5 and 11.2%, respectively at TES and TLS, indicating that APRI contributed to a higher uptake of ^15^N that in deeper soil layer. While when the ^15^N was labeled at 10 cm soil depth, there were no significant (*p* > 0.05) difference of plant ^15^N between APRI and CI treatments. The significantly (*p* < 0.05) higher ^15^NUE by APRI when ^15^N was labeled at 50 cm depth might be explained two reasons: (1) An enlarged root system. Greater dry weight and length density of root were observed under APRI (Table 6). In addition, the root surface area of tomato for nitrogen uptake could be enhanced by APRI as has been indicated in earlier study (Mingo et al., 2004). (2) A high ^15^N availability. APRI maintained the ^15^N in a shallower soil layer relative to CI (Figure 6). Similar result was also reported by Skinner (Skinner et al., 1999) that the alternate furrow irrigation successfully reduced the potential of ${\text{NO}}_{3}^{-}$-N leaching. Moreover, the soil under APRI was proved to have higher microbial biomass (Liu et al., 2015) and accelerated mineralization rate of organic nitrogen (Wang et al., 2010) thus increasing mineral nitrogen available to plants. However, it should be noticed that variation in plant ^15^N could also ascribe to nitrogen isotope fractionation within the plant; for instance, volatilization of NH_3_through stomata preferring ^14^N might result in an increase of plant ^15^N (Wang et al., 2012).

**Table 8:** **The ^15^N uptake by tomato plant under different treatments**.

**Transplant time**	**Treatments**	**Leaf (mg plant^−1^)**	**Stem (mg plant^−1^)**	**Root (mg plant^−1^)**	**Fruit (mg plant^−1^)**	**Whole plant**	
						**Uptake (mg plant^−1^)**	**Utilization rate (%)**
Early summer	APRI10	40.6 ± 1.1a	14.6 ± 0.7a	7.2 ± 0.2a	67.9 ± 2.7a	130.2 ± 4.6a	27.9 ± 1.0a
	CI10	39.6 ± 1.5a	13.7 ± 0.6a	6.8 ± 0.5ab	64.8 ± 1.8a	124.9 ± 4.4a	26.8 ± 1.0a
	APRI50	33.0 ± 2.3c	11.8 ± 0.4b	5.7 ± 0.2c	49.0 ± 1.7c	99.5 ± 4.5c	21.3 ± 1.0c
	CI50	28.6 ± 0.9d	10.2 ± 0.6cd	5.1 ± 0.2d	43.8 ± 2.8d	87.7 ± 4.5d	18.8 ± 1.0d
Late summer	APRI10	37.9 ± 1.2ab	12.0 ± 1.0b	6.7 ± 0.2ab	56.7 ± 2.8b	113.2 ± 3.2b	24.3 ± 0.7b
	CI10	35.3 ± 1.5bc	11.1 ± 0.5bc	6.5 ± 0.2b	55.5 ± 2.1b	108.4 ± 4.2b	23.2 ± 0.9b
	APRI50	33.1 ± 0.6c	9.9 ± 0.2cd	5.9 ± 0.2c	49.6 ± 1.9c	98.4 ± 1.8c	21.1 ± 0.4c
	CI50	28.0 ± 0.9d	9.1 ± 0.3d	5.0 ± 0.1d	46.4 ± 1.6cd	88.5 ± 2.9d	19.0 ± 0.6d
Transplant time (TT)		^*^	^**^	ns	^**^	^**^	^**^
^15^N labeling (NL)		^**^	^**^	^**^	^**^	^**^	^**^
Irrigation pattern (IP)		^**^	^**^	^**^	^*^	^**^	^**^
TT × NL		^*^	ns	ns	^**^	^**^	^**^
TT × IP		ns	ns	ns	ns	ns	ns
NL × IP		^*^	ns	ns	ns	ns	ns
TT × NL × IP		ns	ns	ns	ns	ns	ns

*APRI10 and APRI50 represent that ^15^N is labeled at 10 and 50 cm soil depths respectively under alternate partial root-zone irrigation, CI10 and CI50 represent that ^15^N is labeled at 10 and 50 cm soil depths respectively under conventional irrigation. In the same column, the means followed by the same letter (a, b, c, d) do not differ significantly at 5% level, according to Duncan's multiple range test. Each value is the mean ± SD*.

*, ** *and ns indicate that the experimental treatment has a significant (at 0.05 level) effect, an extremely significant (at 0.01 level) effect, and no significant effect on the organ ^15^N uptake, respectively*.

Under the same irrigation pattern, ^15^N labeled at 10 cm depth significantly (*p* < 0.05) increased the ^15^N amount in the plant parts than that at 50 cm, for both transplant time. Besides, the whole plant under TES had a significantly (*p* < 0.05) higher ^15^N amount than under TLS, when the ^15^N was labeled at 10 cm depth. However, when labeled at 50 cm, the ^15^N difference of the whole plant between two transplant times was not significant (*p* > 0.05). These indicated that the tomato transplanted in early summer could absorb more N from the shallow soil layer. The greatest ^15^N amount for the whole plant (130.2 mg plant^−1^) in this study was detected in APRI10 at TES, and the corresponding ^15^NUE (27.9%) was also the highest.

### ^15^N in soil

**Figure 7:**
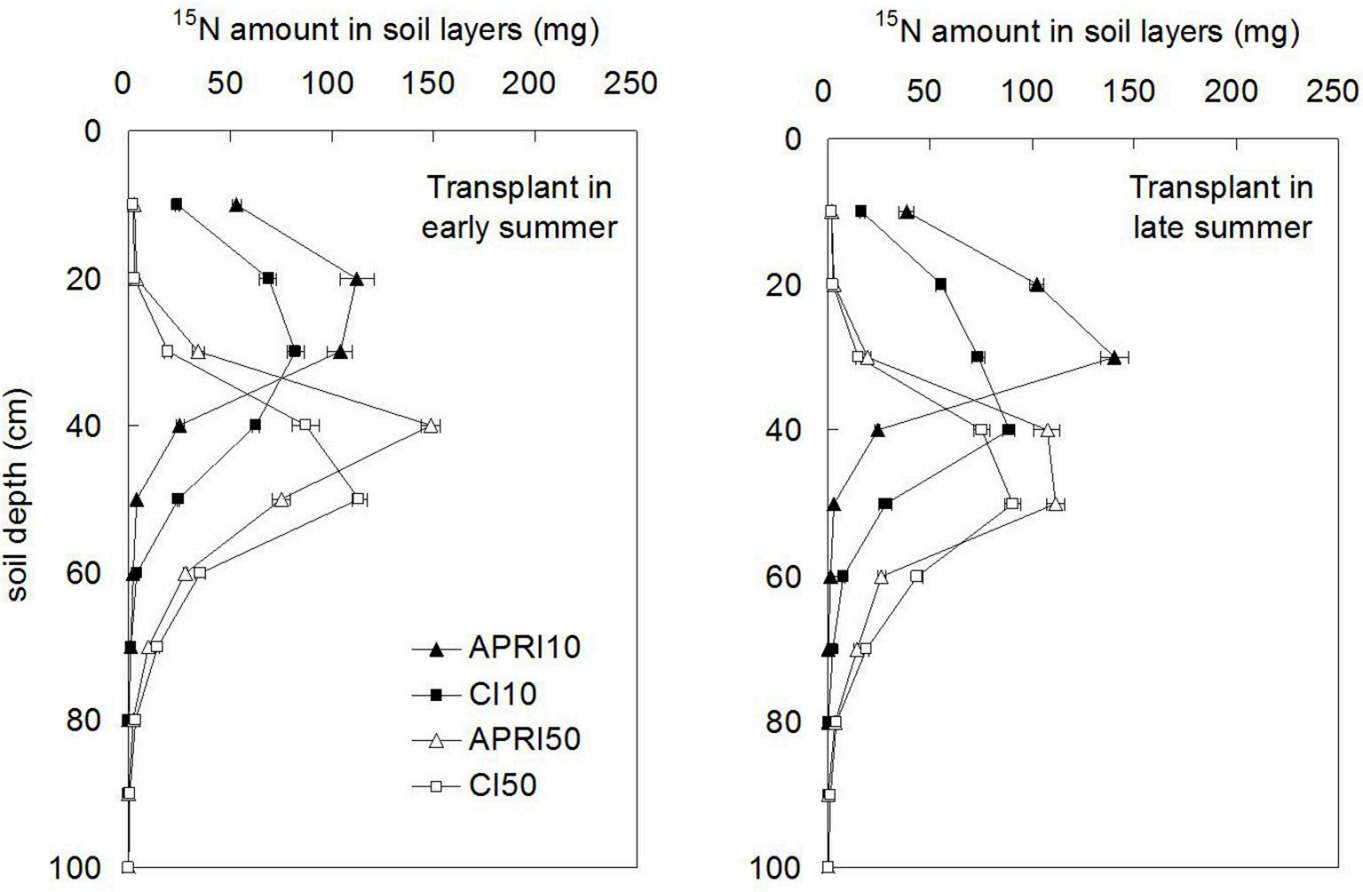
**The accumulation amount of ^15^N in soil layers as influenced by different treatments (APRI10 and APRI50 represent that ^15^N is labeled at 10 and 50 cm soil depths respectively under alternate partial root-zone irrigation, CI10, and CI50 represent that ^15^N is labeled at 10 and 50 cm soil depths respectively under conventional irrigation**. Here, the accumulation amount of ^15^N refers to the amount in each soil column).

Figure 7 displayed the ^15^N distribution in soil layers. The ^15^N labeled at 10 cm depth obviously moved downward with irrigation water, while those labeled at 50 cm depth mostly remained *in situ* or moved upward, only small amount distributed below 60 cm. The peak value of ^15^N under APRI was found in shallower soil layer relative to CI, and this was particularly evident at TES. APRI10 reserved 36.1 and 29.8% of the applied ^15^N in its labeled layer, respectively at TES and TLS, while CI10 reserved only 20.3% and 15.2%. The similar advantage of APRI in reserving ^15^N in corresponding labeled layer could also be obtained through comparisons between APRI50 and CI50. Besides, it was found that TES and TLS differed little on the ^15^N distribution in soil layers.

### ^15^N recovery

The amount of ^15^N recovery for the treatments were ranged from 339.9 to 432.5 mg per soil column, with recovery rate of 72.9–92.8% (Table 9). Correspondingly, the ^15^N loss were from 33.8 to 126.4 mg per soil column, and the loss rate were 7.2–27.1%. The amount of ^15^N recovery in APRI was significantly (*p* < 0.05) higher than in CI when the labeled depth was the same. APRI10 increased the amount of ^15^N recovery by 10.8% (average of TES and TLS) compared to CI10, and APRI50 increased it by 11.7% compared to CI50. Under the same irrigation pattern, the recovery of ^15^N with 10 cm labeled depth were significantly (*p* < 0.05) higher than with 50 cm depth (except CI10 and CI50). Among the different treatments, APRI10 at TES had the highest recovery rate (92.8%) and the lowest loss rate (7.2%) of ^15^N. On the contrary, the lowest recovery rate (72.9%) and the highest loss rate (27.1%) were in CI50, at TLS.

**Table 9:** **The recovery and loss of ^15^N from the soils in 0–100 cm soil layer**.

**Transplant time**	**Treatments**	**Recovery**		**Loss**	
		**Amount (mg)**	**Rate (%)**	**Amount (mg)**	**Rate (%)**
Early summer	APRI10	432.5 ± 11.6a	92.8 ± 2.5a	33.8 ± 11.6e	7.2 ± 2.5e
	CI10	391.9 ± 10.7cd	84.0 ± 2.3cd	74.4 ± 10.7bc	16.0 ± 2.3bc
	APRI50	403.5 ± 11.8bc	86.5 ± 2.5bc	62.9 ± 11.8cd	13.5 ± 2.5cd
	CI50	366.1 ± 11.4de	78.5 ± 2.4de	100.3 ± 11.4ab	21.5 ± 2.4ab
Late summer	APRI10	422.8 ± 9.7ab	90.7 ± 2.1ab	43.5 ± 9.7de	9.3 ± 2.1de
	CI10	379.8 ± 13.6cd	81.4 ± 2.9cd	86.5 ± 13.6bc	18.6 ± 2.9bc
	APRI50	384.7 ± 15.5cd	82.5 ± 3.3cd	81.6 ± 15.5bc	17.5 ± 3.3bc
	CI50	339.9 ± 15.0e	72.9 ± 3.2e	126.4 ± 15.0a	27.1 ± 3.2a
Transplant time (TT)		^*^	^*^	^*^	^*^
^15^N labeling (NL)		^**^	^**^	^**^	^**^
Irrigation pattern (IP)		^**^	^**^	^**^	^**^
TT × NL		ns	ns	ns	ns
TT × IP		ns	ns	ns	ns
NL × IP		ns	ns	ns	ns
TT × NL × IP		ns	ns	ns	ns

*APRI10 and APRI50 represent that ^15^N is labeled at 10 and 50 cm soil depths respectively under alternate partial root-zone irrigation, CI10 and CI50 represent that ^15^N is labeled at 10 and 50 cm soil depths respectively under conventional irrigation. The accumulation amount of ^15^N refers to the amount in each soil column. The ^15^N recovery contains the ^15^N amount absorbed by plant and the ^15^N amount accumulated in 0–100 soil layer*.

*, ** *and ns indicate that the experimental treatment has a significant (at 0.05 level) effect, an extremely significant (at 0.01 level) effect, and no significant effect on the ^15^N recovery/loss, respectively*.

The 4-year case study showed that a 33.3% decrease in irrigation amount led to a 11.0–15.1 kg/ha more recovery of ^15^N (^15^N was originated from double labeled NH_4_NO_3_ with abundance of 10.3%) (Hou et al., 2017). In our study, APRI significantly (*p* < 0.05) increased the ^15^N recovery by 10.2–13.2% but decreased the ^15^N loss by 35.4–54.6% in comparison to CI. The reason might be that APRI could reduce ^15^N leaching, promote ^15^N to move upwards, therefore increase the opportunity for root to capture the ^15^N (Wang, 2014).

Collectively, our results suggested that APRI improved WUE and fruit quality, and noticeably increased the ^15^N recovery, indicating a great potential in reusing the residual fertilizer nitrogen in the soil. In practice, to apply N fertilizer to 50 cm below the ground is difficult, in relation to this, fertigation by using APRI and CI could be an option to evaluate the synergistic effect of spatial and temporal water and N supply to the root zone. This study has three aspects that needed to be noticed or improved: (1) During the experiment process, we have not observed the soil water content continuously using the buried type moisture sensor, this might limit the mechanism analysis of plant water use under APRI. (2) Since we used K^15^NO_3_ as labeling material, the K that added into the soil column might influence the plant growth, yield, particularly the quality. The Na^15^NO_3_ fertilizer, could be considered in similar experiment. (3) In future study, the height of the plastic film used to separate the root system in each soil column should be deeper, in order to achieve better partial root zone drying effect in the deeper soil layers.

## Conclusions

Compared to CI, APRI did not significantly impact the growth and biomass accumulation in the aboveground part of tomato, but it enhanced the root, reflecting by greater length density and more dry mass. APRI produced marginally lower yields, while saved 34.9% of the total irrigation water, and gave a 37.6–49.9% higher WUE relative to CI. In addition, APRI improved the fruit quality, mainly through increasing the contents of soluble solid (by 12.8–21.6% over that of CI) and vitamin C (2.8–12.7%), and the ratio of sugar/acid (3.5–8.5%). The ^15^NUE of tomato in APRI was higher than that in CI, which was more evident when the ^15^N was labeled at the soil depth of 50 cm. The significant (*p* < 0.05) ^15^N recovery increase of 10.2–13.2% and ^15^N loss decrease of 35.4–54.6% were found for APRI compared to CI. Surprisingly, in our study, different transplant time showed little differences in growth, yield and quality of tomato, except that transplanting in late summer caused a delay in the first harvest of tomato and increased the volume of single fruit significantly (*p* < 0.05). We concluded that an enlarged root system and a high ^15^N bioavailability under APRI might have contributed to the higher ^15^NUE of tomato.

## Author contributions

In this study, author MH wrote the main manuscript. QJ gave the valuable guidance for the experiments. XL, JL, and HZ analyzed the data. In addition, YG helped to modify the manuscript.

### Conflict of interest statement

The authors declare that the research was conducted in the absence of any commercial or financial relationships that could be construed as a potential conflict of interest.
